# Current-Mode Self-Amplified CMOS Sensor Intended for 2D Temperature Microgradients Measurement and Imaging

**DOI:** 10.3390/s20185111

**Published:** 2020-09-08

**Authors:** Patrick M. Santos, Davies W. L. Monteiro, Luciana P. Salles

**Affiliations:** 1 Department of Electrical Engineering, DEE, Federal Center for Technological Education of Minas Gerais-CEFET-MG-Av. Amazonas 7675, Nova Gameleira, Belo Horizonte-MG 31510-000, Brazil; 2 Department of Electrical Engineering, DEE/PPGEE, Federal University of Minas Gerais-UFMG-Av. Antônio Carlos 6627, Pampulha, Belo Horizonte-MG 31270-010, Brazil; davies@ufmg.br (D.W.L.M.); luciana@cpdee.ufmg.br (L.P.S.)

**Keywords:** temperature microgradient, thermal image, uncooled temperature sensors, current-mode circuits, APS array, CMOS technology

## Abstract

This paper presents the design of a current-mode CMOS self-amplified imager operating in dark conditions, for thermal imaging, which provides an innovative solution for precision thermal contact mapping. Possible applications of this imager range from 3D CMOS integrated circuits to the study of in-vivo biological samples. It can provide a thermal map, static or dynamic, for the measurement of temperature microgradients. Some adaptations are required for the optimization of this self-amplified image sensor since it responds exclusively to the dark currents of the photodiodes throughout the array. The sensor is designed in a standard CMOS process and requires no post-processing steps. The optimized image sensor operates with integration times as low as one μs and can achieve both SNR and dynamic range compatible to those of sensors available on the market, estimated as 87dB and 75dB, respectively; noise equivalent temperature difference can be as low as 10mK; and detection errors as low as ±1%. Furthermore, under optimal conditions the self-amplification process enables a simple form of CDS, enhancing the overall sensor noise performance.

## 1. Introduction

### 1.1. Thermal Sensor Concept

Many contributions to temperature sensors and temperature imagers are available in the corresponding technical literature. Regarding the uncooled thermal image sensors, some papers present contributions considering area reduction, enhancement of responsivity and sensitivity, signal readout process, image processing, power consumption and noise equivalent temperature difference (NETD) [1,2,3,4,5,6,7,8,9,10,11,12,13,14,15,16,17,18,19,20]. It is desirable to have these sensors integrated into standard CMOS technology to reduce the costs and to facilitate embedding the interface and the readout electronic circuitry [1,2,4,5,7,10,11,12,17,18]. Among those sensors having some degree of integration, none can be fabricated without changes to the standard CMOS process: breaking some design rules, adjusting process steps/sequence or by performing post-processing steps [1,2,4,5,7,10,11,12,17,18]. Besides the uncooled thermal image sensors, there have been developments in the cooled infrared sensors, even though they are more expensive and usually not integrated with CMOS signal processing circuits [21,22,23,24,25,26]. These thermal image sensors have been developed to acquire images of objects at a distance, that is, not in contact with the sensor, and based on microbolometers, quantum-well detectors, and materials other than silicon, resulting in imagers with reduced resolution compared to cameras for the visible spectrum.

Most temperature sensors are available for MEMS -based microscale measurements, and not all of them have the desired integration with the CMOS technology, to feature essential functions such as amplification and signal conditioning [11,15,17,27,28,29,30,31,32,33,34,35,36]. Furthermore, there have been developments in the nanoscale thermal sensing [37,38,39,40,41]. Some of these nanoscale sensors are used in contact with the measured sample [27,28,31,33,34,36,37,38]. Interestingly, even though they have been developed to sense temperature, they can also yield images, either through direct contact with the measured sample [33] or at a distance [11,15,17,33,35].

This paper presents an image sensor based on a standard CMOS process with 20μm×23μm pixel size for temperature readings up to $80\;{}^{\circ }C$ (studies were carried out until $100\;{}^{\circ }C$, but the checking process was made up to the $80\;{}^{\circ }C$ limit only). This temperature range is compatible with some potential applications [42,43,44,45,46,47,48,49,50,51]. These readings are obtained with the sensor in direct contact with the body or fluid and can provide 2D images of the sample in 1μs. This array of pixels also allows the registration of temperature gradients. The developments were performed based on previously published work, in which we introduced a topology for a current-mode CMOS imager, in the visible spectrum, with advantages such as high signal swing and flexibility of operation when compared to voltage-mode solutions for the same feature size [52,53]. This pixel area was made arbitrarily big to afford the proof of concept for the visible light imaging, being the thermal imaging an application that came up later as an additional possibility. This pixel size is suitable for the investigations of the temperature dependency of human cells growth and activity [43,44,51] or gas thermal behavior [17,54].

Since the sensor was designed using standard CMOS technology, it can be rather inexpensive when in mass production, scalable and reliable, and demands no additional post-processing. The design results show promising advantages compared to other contactless or contacting thermal imagers since the sensor can be—(i) customizable: pixel dimensions can be tailored to several types of structures or applications with different spatial distribution of temperature; (ii) as sensitive as the others, regarding the NETD [2]; (iii) cheaper; (iv) faster, since images can be registered with integration times of only 1μs or less (depending on the biasing conditions); (v) small, and they can be in direct contact with the system to be monitored and packaged together; and (vi) self-amplified, that is, the pixel matrix senses the signal and also intrinsically amplifies it.

It is important to emphasize that all of the aforementioned sensors have a wide range of applications and temperature sensing capabilities. Different time responses, detectivity, temperature range, spatial resolution, and frame rates have been reported among them.

The proposed sensor can be used to constantly show and monitor temperature, for instance, to: (i) perform reliability tests of integrated ICs [55,56,57,58,59,60]; (ii) control the temperature of microfluidic systems [61,62]; (iii) control the temperature in microbiological experiments [42,43,44,45,46,48]; (iv) enhance Chemistry and Physics-related experiments [49,51,63]; and (v) help other types of studies [50,64,65,66,67].

In this paper, we briefly discuss basic electronic schematics and present details regarding the operation of visible light image sensors operating in dark conditions. Design aspects, operation, and control are described in sequence. Section 3 presents and discusses the results regarding sensor linearity, frequency response, and noise performance.

### 1.2. Considerations

The thermal image sensor presented here is intrinsically an image sensor for the visible spectrum [52,53]. The concept is to use the current-mode image sensor as its own signal amplifier using a particular arrangement of transistors and operation (detailed in Section 2). The pixels of the matrix perform two different roles: control and scene. Once operating as scene pixel, its function is to provide the current signal to the output, and the intensity of this signal is set by the control pixel. The sensor scans all the matrix, in the readout clock frequency, pixel by pixel, in such a way that just one pixel at a time will be the control pixel while the others are the scene pixels. The sensor signal output is the sum of all the scene pixels output currents, and this sum will vary based on the information coming from the selected control pixel.

Summarizing, the sensor operates in integration mode and acquires information during the integration time. After this, it enters in the readout mode while the pixels are scanned, and one by one, they act as a control pixel, proving its information (output voltage) that commands the rest of the matrix pixels (scene pixels) to output a current signal. This signal is proportional to the output voltage of the selected control pixel.

In an image sensor for the visible light spectrum, the information comes from incident light. In this application, the information comes from a body (another chip, for instance) in physical contact with it. The photodiodes sense the changes in temperature throughout the body that is in contact with them. Different pixels can read different temperatures if there is proper thermal contact between the body and the sensor.

The results in this paper come from simulations of a sensor design in a 0.35μm CMOS process (with four metal layers and maximum supply voltage of 3.3V). Furthermore, we expect that the current-mode operation will produce higher output signal swings than the voltage-mode output signals when both are compared to their respective noise floor. The expectation comes from the fact that ICs with smaller feature sizes operate with lower voltages than the ones with bigger feature sizes, regarding the voltage-mode. In the current-mode, once the current densities design rules are observed, the signal swings can be kept constant even with smaller feature sizes. This increased signal swing will lead to enhanced dynamic range (DR) and signal-to-noise ratio (SNR).

It is also important to emphasize that silicon is a good thermal conductor, and the pixel thermal crosstalk must not be neglected. We performed an initial study just to ensure that there will be less thermal resistance between the pixel and the sensed body than between the pixel and its neighbors, considering the materials available at the CMOS process used and the thermal interface materials (TIMs) available on the market. Besides the enhancement of the vertical thermal conductance with proper contact interface with a substance between the chip surface and the object to be sensed, a good intra-matrix thermal insulation can be better ensured in processes where shallow or deep trench isolation is available. In this paper, we make no further considerations regarding the best solution to produce such isolation or optimal thermal contact, such as the application of TIMs with high thermal conductance. We focus on the concept of the thermal sensor and image formation.

At last, we highlight that the need for a TIM applies for solid objects only, not applicable to gases or fluids. And, in the use of a TIM with a solid object, the material choice and the application method might interfere in the sensing capability, also implying in possible lateral heat flow inside the TIM, that must be taken into account in order to have the desired thermal contact.

All the results that are presented in this paper were obtained considering a perfect thermal contact between the body being sensed and the sensor array.

## 2. Materials and Methods

### 2.1. Analog Signals Analysis

**Figure 1:**
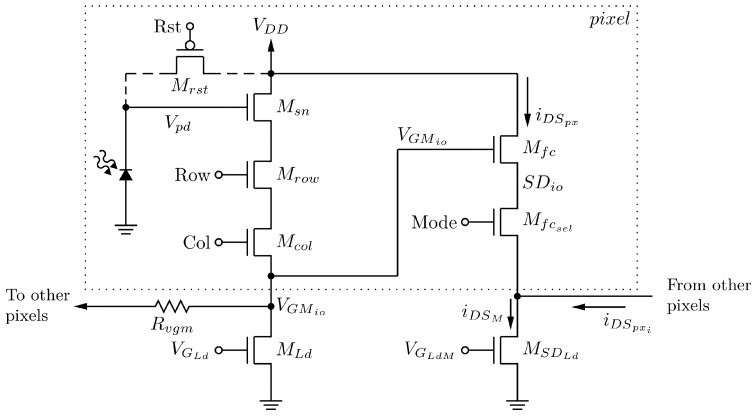
Simplified schematics of self-amplified matrix pixels and its active loads.

Figure 1 shows the sensor pixel schematic, that has been developed in related prior work [53].

**Figure 2:**
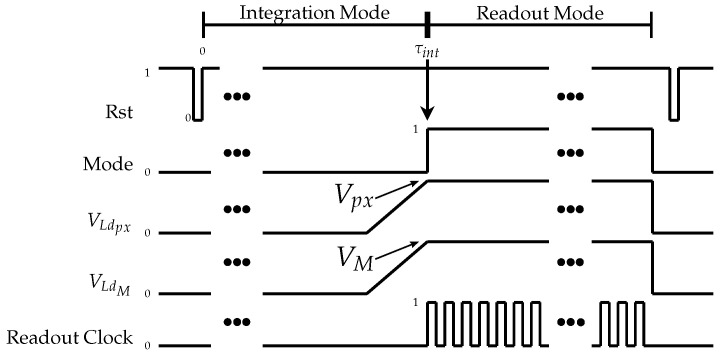
Signals used to control the matrix operation.

It is noteworthy that the transistors $M_{Ld}$ and $M_{{SD}_{Ld}}$ are outside of the pixel area and are shared by all of the pixels since they are operated in a very particular way, as we describe next. The transistor $M_{{SD}_{Ld}}$ (in Figure 1) has its source attached to the ground for simplicity. Its source is where any other readout circuitry can be connected for any auxiliary signal processing: from filtering to digital conversion. In Figure 2, it is possible to notice two different modes of sensor operation: the integration and the readout.

The mode of operation is set by the signal Mode that turns transistor $M_{{fc}_{sel}}$ on and off. When it is turned off (Mode=0): (i) the pixels are reset to the value of $V_{DD}$ because of the PMOS reset transistor; (ii) the matrix will be in integration mode, that is, sensing the temperature and (iii) it will take $\tau_{int}$ seconds to integrate the signal: the dark current is discharging the photodiode internal capacitance and providing the voltage drop that will be read in the readout mode.

The readout mode is set with Mode=1. To control the active loads for the sense amplifier ($M_{sn}$) and the modulation amplifier ($M_{fc}$), we adjust the values of the biasing voltages $V_{{Ld}_{px}}$ and $V_{{Ld}_{M}}$, respectively. All pixels share both active loads, although the connections coming from all pixels to the drain of $M_{{SD}_{Ld}}$ are not highlighted in Figure 1.

### 2.2. Modes of Operation

**Figure 3:**
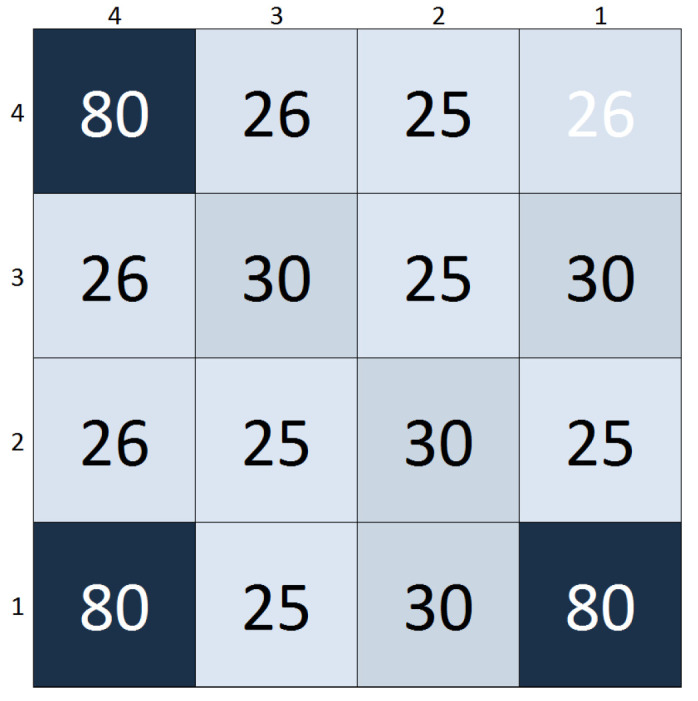
Temperature map for matrix operation test.

**Figure 4:**
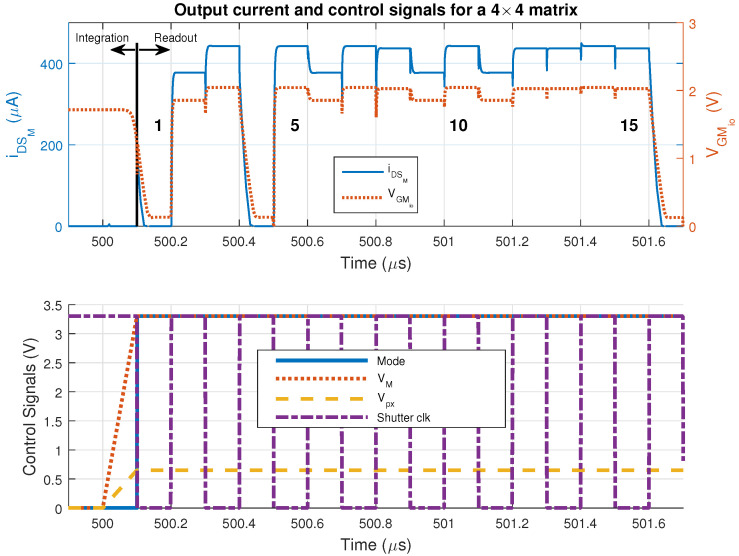
Output current $i_{{DS}_{M}}$ and control signals: $V_{px}=0.65\;V$, $V_{M}=3.3\;V$, $\tau_{int}=500\;\mu s$.

Figure 3 and Figure 4, respectively, illustrate the two modes of operation and their related signals for a 16 pixels (4×4) (to conveniently show the matrix operation and provide de details in Figure 4. The choice of such high $\tau_{int}$ value is only to demonstrate how versatile this sensor can be) matrix (pixels number 1, 5, 10 and 15 are identified in Figure 4). Up to 500.1μs, the sensor is acquiring information, and Mode=0 and the readout clock is kept steady at 3.3V until the same time. At the end of integration, Mode goes to 1 and $V_{{Ld}_{px}}$ and $V_{{Ld}_{M}}$ complete their transient, putting the sensor in the readout mode. The matrix current ($i_{{DS}_{M}}$) in this example is the sum of 16-pixel currents ($i_{{DS}_{px}}$), and it is zero during the integration time. The output is only available in the readout mode.

The integration time $\tau_{int}$ was arbitrarily set to 500μs, started after a delay of 100ns (to avoid power-up transients). The readout clock frequency was set to 10MHz. The higher the temperature, the higher the dark current and the lower the cathode voltage at the end of $\tau_{int}$. Therefore, the higher the temperature, the smaller the output current $i_{{DS}_{M}}$. For instance, the mean temperature at pixel 4 (row 1, column 4), which becomes the control pixel at 500.4μs, is higher than the mean temperature at pixel 5. The impact in the $i_{{DS}_{M}}$, sourced by the $V_{{GM}_{io}}$ provided by pixel 4, is lower than the one sourced by the pixel 5 output (row 2, column 1).

The ramps on Figure 2 help to charge the capacitances of $M_{{SD}_{Ld}}$ smoothly, thus avoiding high turn-on peak current output by the sensor at the beginning of the readout mode. Figure 4 shows a peak like this at 500.1μs.

During the readout mode, each pixel assumes the control of the whole matrix (control pixel) once selected (transistors $M_{row}$ and $M_{col}$ are on). The output voltage of the selected control pixel sets the output current of the other ones (scene pixels). This voltage is $V_{{GM}_{io}}$ and is now available to all the scene pixels. Each one of them will source its $i_{{DS}_{px}}$ to the $M_{{SD}_{Ld}}$ transistor forming the $i_{{DS}_{M}}$ current. The matrix current $i_{{DS}_{M}}$ is a larger copy of the $i_{{DS}_{px}}$ of the control pixel during the readout clock period. This summing process, namely the matrix amplifier, is part of the sensor concept. In other words, this is a self-amplified 2D array.

It is possible to predict that the sensor could output: a VGA image with 30 fps considering the presented simulation numbers and a 10MHz readout clock. Serial communication with the controller or HD images at 30 fps, if an 8-bit parallel communication bus is implemented, considering an integration time of 1μs.

There is a selection logic (not detailed in this paper) that operates at the frequency of the readout clock and will select a pixel at a time, finishing the electronic shutter operation.

### 2.3. Pixel Design

**Figure 5:**
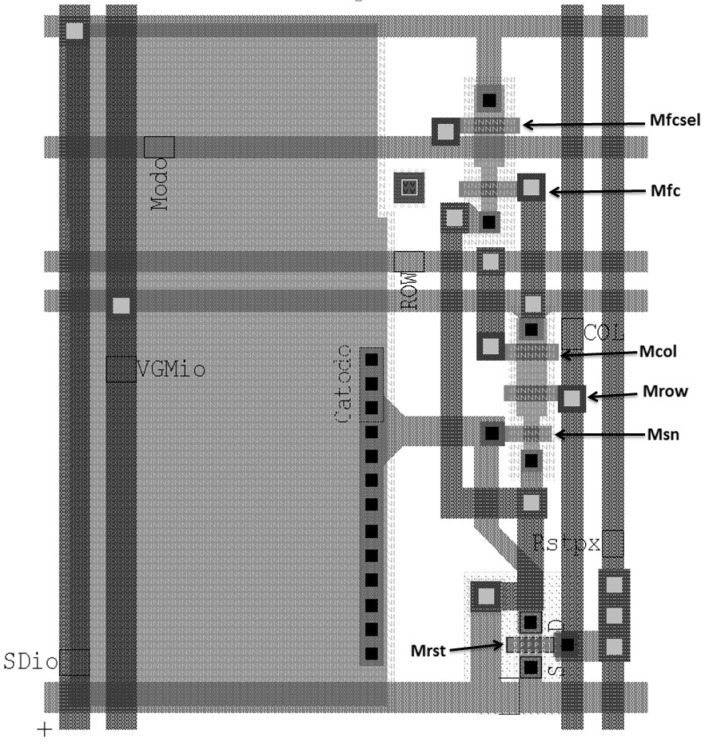
The layout of the pixel in 0.35μm process.

The simulations were based on a pixel layout designed on a 0.35μm process with ***BSIM3v3*** transistor models. Figure 5 shows the layout without two thermally connected metal layers that lie above the pixel. Since the CMOS process in use has four metal layers, the last two were used to cover the pixel area and improve thermal contact with the photodiode. Since they hide the pixel design, they are not displayed in Figure 5, covering its whole area and improving thermal contact. The resistor $R_{vgm}$, presented at the schematic, models the interconnection resistance of the metal layer that connects adjacent pixels. Although the photodiode area was intentionally made large (compared to pixels in high-resolution cameras for the visible spectrum)—≈$220\;\mu m^{2}$, it is comparable to other uncooled thermal imagers [1,2,3,4,10]. The choice of a PMOS as a reset transistor increases the pixel size but allows maximum voltage reference value at the cathode, extending the signal swing. The pixel dimensions are 20μm×23μm (L × H), leading to a fill factor of 45.8%. All transistor channel lengths have been dimensioned to 0.5μm, considering the technology feature size while keeping some safe margin regarding process variation. Transistors $M_{fc}$ and $M_{sn}$ have a unitary aspect ratio. The aspect ratio of $M_{row}$, $M_{col}$, and $M_{{fc}_{sel}}$ is 2. For $M_{rst}$, it is 1.5.

(1) $$\;A_{1}\;=\frac{V_{{GM}_{io}}}{V_{pd}}=\;\frac{g_{m_{sn}}R_{1}}{1+g_{m_{sn}}R_{1}}$$

(2) $$\;A_{2}\;=\frac{V_{S\;D_{io}}}{V_{{GM}_{io}}}=\;\frac{g_{m_{fc}}R_{2}}{1+g_{m_{fc}}R_{2}}$$

(3) $$\;R_{1}=r_{o_{sn}}||\left(r_{o_{Ld}}+R_{{on}_{row}}+R_{{on}_{col}}\right)||\frac{1}{g_{{mbs}_{sn}}}$$

(4) $$\;R_{2}=r_{o_{fc}}||\left(r_{o_{SDLd}}+R_{{on}_{fcselt}}+R_{med}\right)||\frac{1}{g_{{mbs}_{fc}}}$$

Thermal imaging is treated here as a particular case of imaging formation since the temperature rise yields a higher dark current in the photodiode. Also, pixels with different mean temperature rise will experience different dark currents, thus providing different values for $V_{{GM}_{io}}$. For the pixel circuit, the temperature rising is equivalent to the photodiode receiving more light.

The temperature produces an increment in (dark) photocurrent generally smaller than the one achieved with the light impinging the photodiode. The pixel biasing is responsible for adjusting the pixel gain and must be chosen according to the application. More specifically through the parameters: (i) the sense amplifier transconductance and output resistance ($g_{m_{sn}}$, $r_{o_{Ld}}$), and (ii) the modulation amplifier transconductance and output resistance ($g_{m_{fc}}$, $r_{o_{SDLd}}$). Equations (1) and (2) indicate that the biasing current set by $V_{{Ld}_{px}}$ and $V_{{Ld}_{M}}$ to the sense amplifier and modulation amplifier, respectively, will dictate the values of $g_{m_{sn}}$, $g_{m_{fc}}$, $r_{o_{Ld}}$ and $r_{o_{SDLd}}$, thus interfering in the overall transconductance pixel gain. Therefore, those two voltages will set the amount of $i_{{DS}_{px}}$ (and also $i_{{DS}_{M}}$) relative to a $1\;{}^{\circ }C$ temperature difference, that is the pixel detectivity. In those equations: $A_{1}$ is the gain of the sense amplifier and $A_{2}$ is the gain for the modulation amplifier.

As mentioned before, the connection between the source of $M_{{SD}_{Ld}}$ and ground is external to the matrix and any post readout circuit should be connected there. Thus, the circuit impedance of appears in series with the amplifier and, the higher the impedance, the smaller the output current.

Both amplifiers roughly have the same transfer function. Nevertheless, regarding the sense amplifier, the output/input voltage relation is more important. Regarding the modulation amplifier, we are interested in the relationship between the output current and input voltage. Increasing values of both biasing voltages will decrease the load resistance of the respective amplifier. With the help of Figure 1 and using Equations (1) and (2), one can write the current gain for the modulation amplifier:

(5) $$\frac{i_{{DS}_{px}}}{V_{{GM}_{io}}}\;=\;A_{2}\times \frac{1}{R_{2}}\;=\;\frac{g_{m_{fc}}}{1+g_{m_{fc}}R_{2}}$$

Regarding the sense amplifier, the higher the value of $V_{px}$, the higher the amplifier current. The higher the current the smaller the value of $R_{1}$, the gain (usually the transconductance increase does not compensate for the changes in the output resistance [68]) and also the $V_{{GM}_{io}}$ swing (it sets the drain-source voltage of $M_{Ld}$). Besides that, it sets the $M_{sn}$ transistor into the triode operation region (or even to a moderate inversion state of operation) and promotes higher values of transistor transconductance. Adopting this same analysis to the modulation amplifier, the higher the value of $V_{M}$, the higher the current gain.

### 2.4. Thermal Analysis of Sensor Operation

**Figure 6:**
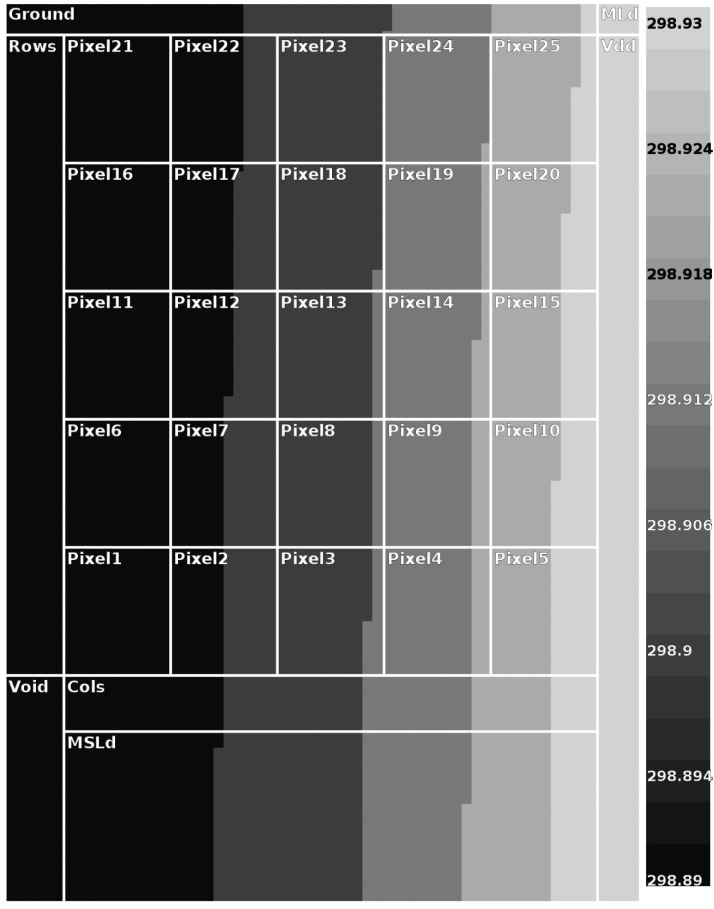
Continuous operation mode.

**Figure 7:**
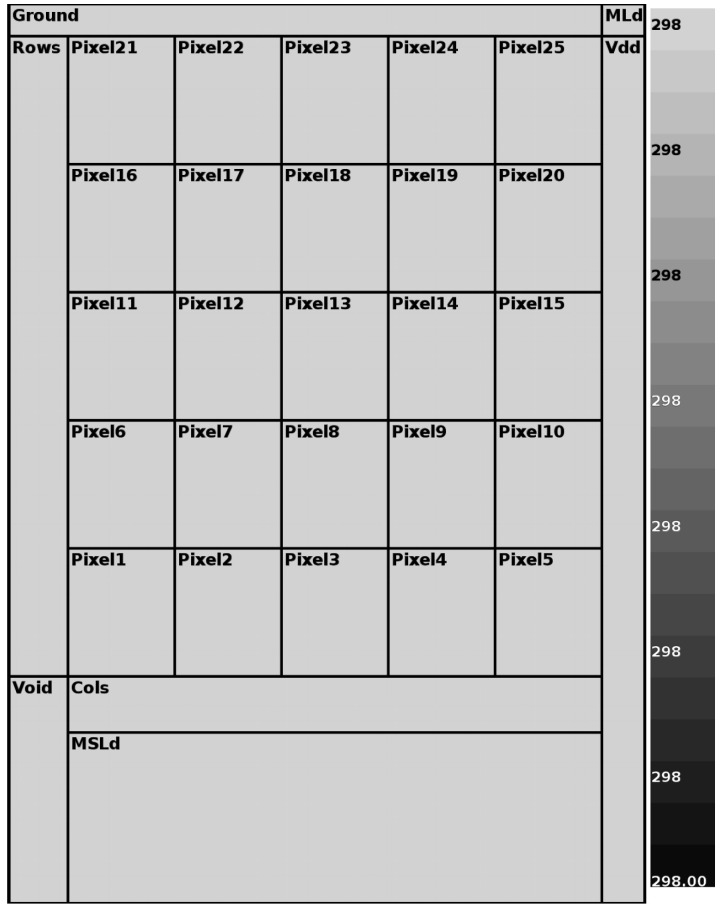
Scheduled operation mode (1ms).

The thermal behavior of the sensor operation was investigated to assure that there is no impact on temperature rising on pixels. That is, the sensor must be able to perform its tasks without generating any noise or interference on the information received from the object being “probed”. This process was investigated considering two situations—(i) the sensor would be in continuous integration/reading process. In other words, after 10μs of integration time, the sensor would start the readout mode, going back to the integration mode and so on; (ii) the sensor would output 30 frames during one second (one sample of one second with 30 frames) and then rest for 1ms before the next sample. Regarding the sensor operation, without any “bodies to be viewed”, temperature maps are generated with the help of the HotSpot methodology [69]. This investigation was done to guarantee that the sensor itself would not produce an excess of temperature that would interfere in its reading. Figure 6 and Figure 7 display the output maps from HotSpot simulation.

These figures show the positions of transistors $M_{Ld}$ and $M_{{SD}_{Ld}}$ which are shared among all pixels, as shown in Figure 1. Since these transistors are not within the array, and considering layout choices to guarantee the highest possible thermal insulation in a CMOS process, it is expected they will be kept at a more constant and lower temperature than the pixels. The $M_{{SD}_{Ld}}$ was specially made with larger metal tracks to ensure that there would be no hot-spot caused by the current flow from it.

A 5×5 (the highest achieved for a good evaluation and maximum for the sake of computational economy) example sensor was used while keeping the most power-consuming matrix elements (the active loads and row/column, ground, and $V_{DD}$ buses) in the IC floor plan. We used the layout that was also conceived to correctly estimate the power and areas for (i) columns and rows; (ii) supply voltage rail; and (iii) metal tracks responsible for output current distribution throughout the matrix.

Although $25\;{}^{\circ }C$ (298K) is the lowest considered temperature, and the thermal noise is higher at $80\;{}^{\circ }C$ operation, the simulations were implemented at $25\;{}^{\circ }C$. In this situation, as stated before when analyzing Figure 4, the smaller the temperature, the higher the current sensor $i_{{DS}_{M}}$, and the higher the power consumption and matrix self-heating. Because of that, in a percentage analysis, any temperature rise around $25\;{}^{\circ }C$ is more significant.

The continuous mode of operation produced a steady-state maximum temperature difference between parts of the matrix of 36mK. However, by outsourcing the scene with 30 frames and resting for 1 ms, the temperature differences are lower than 1mK. So it is possible to operate the sensor in such a way that it can produce usable output without significant interference. The full details are in Table 1.

## 3. Results and Discussion

We have used foundry models and the pixel from Figure 1 to simulate a matrix with 16lines×16columns (this matrix size was the intended one for the applications mentioned in Section 1.1). The main goals of the simulations were:

1. To assess the influence of the matrix biasing on the linearity and sensitivity of the sensor.
2. To evaluate the best and worst case for detection errors.
3. To evaluate the behavior of the sensor in the frequency domain and its noise performance.

### 3.1. Linearity, Error and Sensitivity

As presented in a previous work [53], the effective current, or $\Delta i_{{DS}_{M}}$, is defined as the difference between the $i_{{DS}_{M}}$ registered in the dark and the $i_{{DS}_{M}}$ registered with the current control pixel. In the case of the thermal imager, the reference condition will be the room temperature (calibration procedures were designed but are not the focus of this paper). This current difference will be the actual output of the self-amplified matrix. The readings were performed in the linear portion of the transconductance characteristic of the $M_{fc}$ transistor inside every pixel [53]. If $i_{{DS}_{px}}$ responds linearly to the $V_{{GM}_{io}}$ from the control pixel, so will $i_{{DS}_{M}}$ and $\Delta i_{{DS}_{M}}$. The value of $V_{px}$ (Figure 2) must be controlled, since it sets the biasing of the sense amplifier, hence changing its output voltage, impacting the maximum output current, as explained in the previous section. This process must be done, not only because of detection errors but also because of noise generation and thermal dissipation. The results presented are for $V_{px}=1.3\;V$.

Regarding this thermal imaging application, it is necessary to obtain a higher gain when compared to visible-light imaging, due to the temperature rise not producing photodiode current increments as high as the light does. In that way, the other biasing voltage was set to $V_{M}=3.3\;V$.

**Figure 8:**
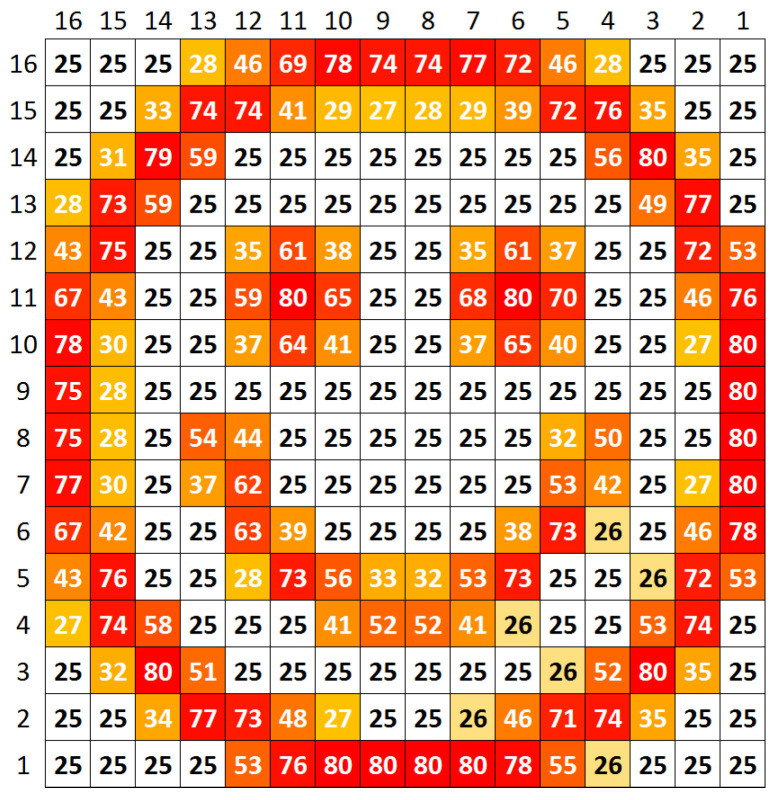
Mean absolute temperature.

**Figure 9:**
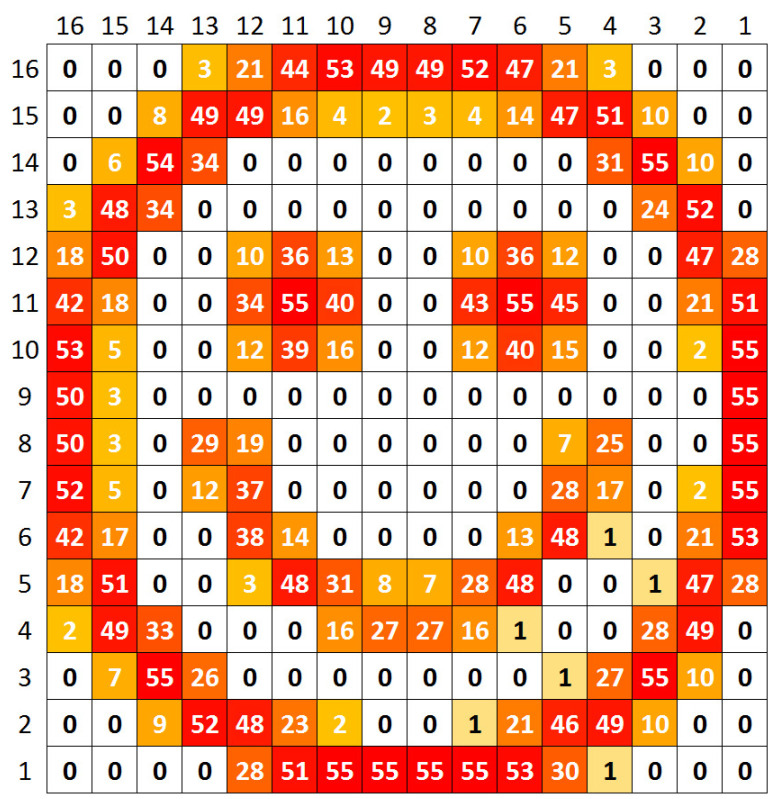
Mean temperature rise.

The test procedure was the following—a 2D array of the described pixels was exposed to a heat pattern in the shape of a smiley face, with pattern lines exhibiting various temperatures above the room temperature, which we arbitrarily set as $25\;{}^{\circ }C$. So each pixel minimum temperature is $25\;{}^{\circ }C$. The current mean temperature at each pixel can be seen in Figure 8. The map in Figure 9 presents the mean temperature rises above the room temperature. The values in both figures are in Celsius. The simulations were centered in a temperature range (as mentioned before, simulations were performed with temperatures up to $100\;{}^{\circ }C$) that covers most of the applications mentioned in Section 1.1.

Based on the aforementioned pixel layout, the area being sensed is 320μm×368μm. By Figure 8 and Figure 9, one can see that there are spatial temperature gradients ranging from 0 to $5.5\;/\;{}^{\circ }C\;\mu m$. With smaller pixel sizes, the same area could be covered with more pixels leading to different spatial temperature gradient distributions.

**Figure 10:**
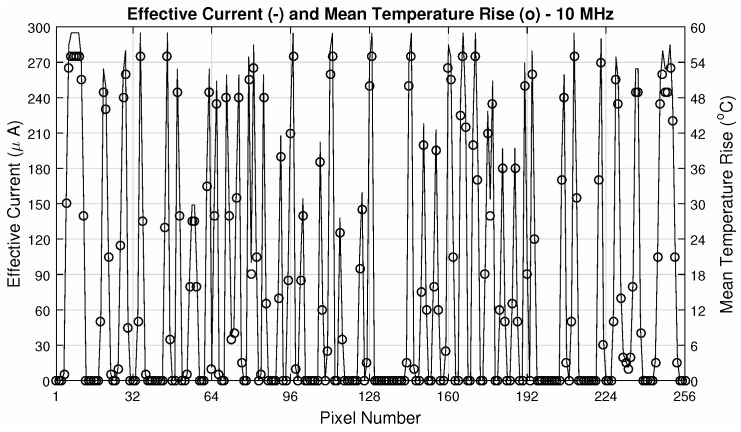
Effective current (spline) and temperatures (markers) for 10MHz readout.

**Figure 11:**
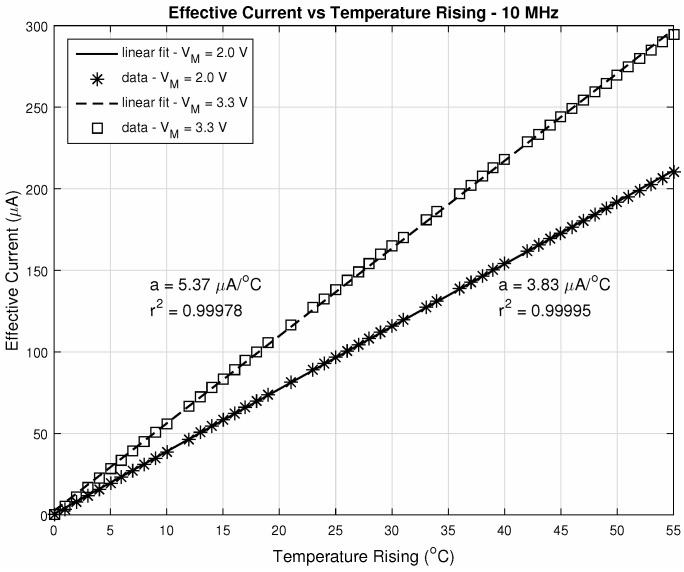
Sensitivity (response characteristic) at 10MHz complete readout.

**Figure 12:**
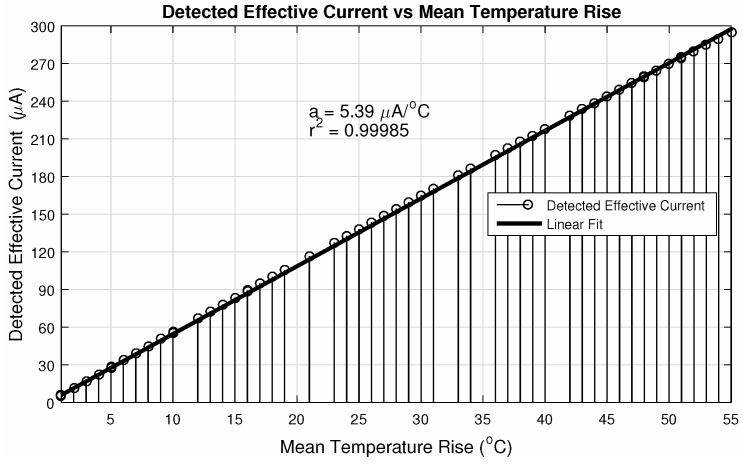
Actual effective current evolution with temperature for 10MHz readout.

Operating parameters are—an integration time of $\tau_{int}=10\;\mu s$ and readout frequency of 10MHz. The pixel # 1 is the one with (row/column) address equals to (1,1) (bottom right of the matrix) and the # 256 with (16,16) (top left of the matrix), being the matrix scanned throughout the columns of each line, until the last one. The $\Delta i_{{DS}_{M}}$ was calculated as described before: with the difference between the matrix current $i_{{DS}_{M}}$ at room temperature ($25\;{}^{\circ }C$) and the actual $i_{{DS}_{M}}$ due to the control pixel being read. These parameters provide a good enhancement against noise, since fixed-pattern noise is systematic and will be removed in this operation. In other words, the $\Delta i_{{DS}_{M}}$ calculation is a simple version of a Correlated Double Sampling method (***CDS***). Figure 10 exposes the $\Delta i_{{DS}_{M}}$ and the corresponding temperature of these 256 pixels. Figure 11 and Figure 12, respectively, bring the sensor response characteristics, expected and registered. The values for sensitivity are slightly different because in Figure 11 the calculation was performed using only the different available temperature values, and the one in Figure 12 was calculated using all the 256 current samples.

The sensitivity for $V_{M}=3.3\;V$, as shown in Figure 11, is the highest possible value considering the proposed configuration together with this level of linearity. The linearity can be enhanced with lower values for this biasing voltage, which is the case for $V_{M}=2.0\;V$, in the same Figure. Since it is possible to work with almost 40% more sensitivity, with only 0.02% less linearity, it is again justifiable the proposed value of 3.3V. It is important to highlight that we adjust the modulation amplifier bias to set the desired error level and sensitivity.

**Figure 13:**
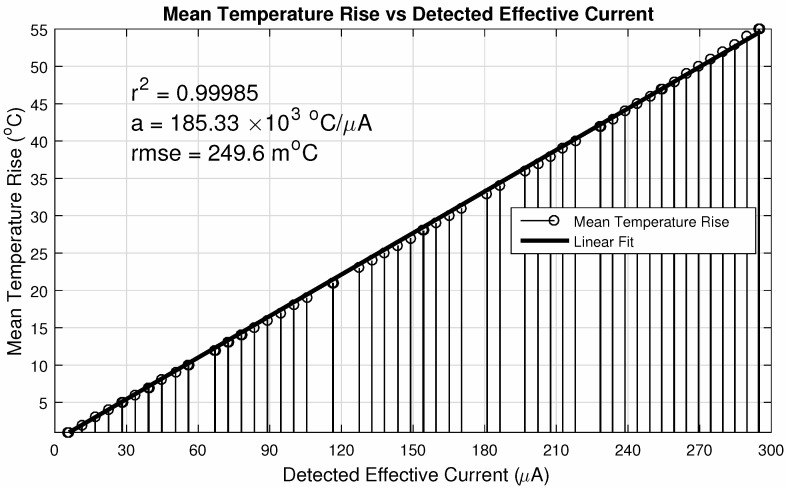
Detected temperature concerning $\Delta i_{{DS}_{M}}$.

Since the integration time is quite short, not allowing for significant discharge of the photodiode internal capacitance with the reverse current, the photodiode voltage will be close to the reset voltage and will stay close to it. This allows us to analyze the current as dependent only on the temperature and, as a consequence, to retrieve the last from the former. Figure 13 displays the temperature obtained from the $\Delta i_{{DS}_{M}}$.

**Figure 14:**
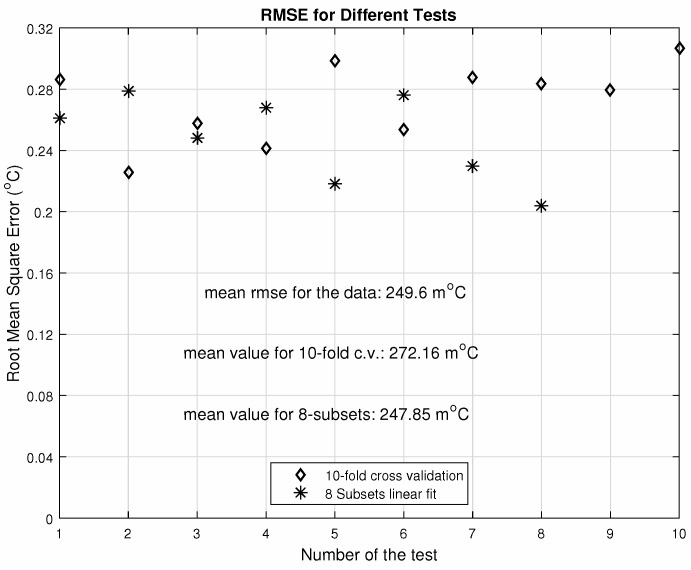
Root mean square error variation in the statistical validation.

Figure 14 shows different calculations of the root mean square error, which has been chosen as the error evaluation parameter since it can be statistically validated. Its value was approximately $0.25\;{}^{\circ }C$, which is quite low compared to the range of detection (0.45%). The statistical analysis includes the cross-validation of the datasets. The simulation process is very time consuming and demands a considerable processing effort, so we chose the k-fold cross-validation of the models as the main statistical validation method. Furthermore, the dataset was divided into eight subsets (the nearest integer of the 10-fold) for linear fits into each subset. We have compared the root mean square error variation for both analyses—10-fold cross-validation and the 8 subsets division. Both analyses resulted in mean values of the root mean square error close to that of the whole dataset.

**Figure 15:**
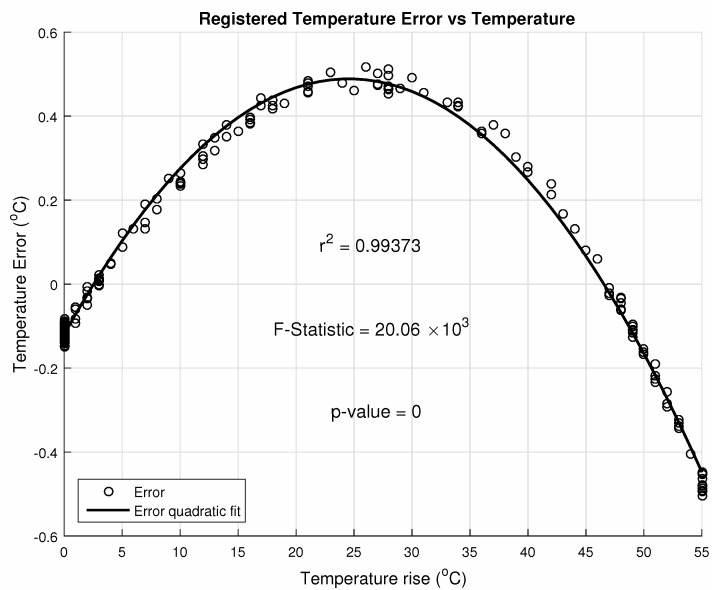
Reading error dependence on pixel temperature at 10MHz.

The error is dependent on the temperature, and Figure 15 shows its evolution in the observed intervals; their relationship is statistically significant.

**Figure 16:**
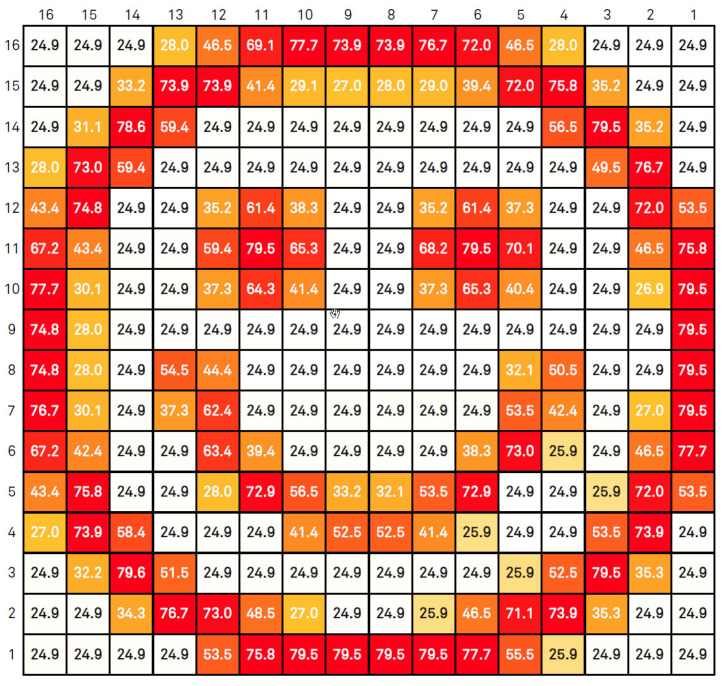
Detected absolute temperatures.

**Figure 17:**
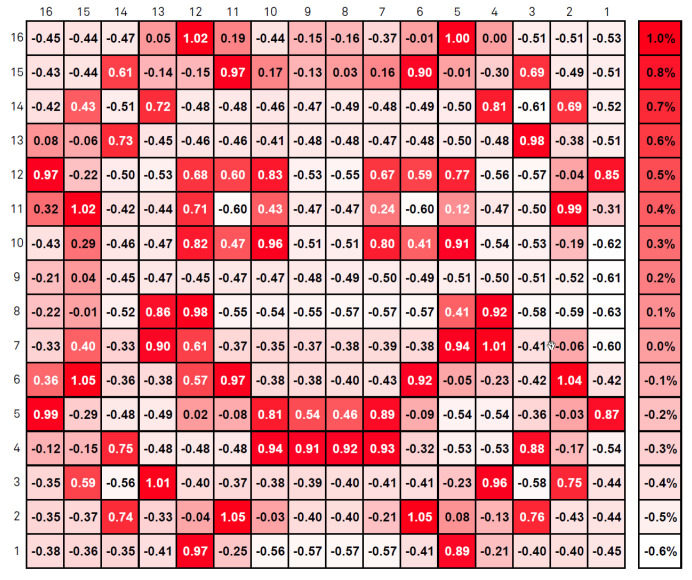
Percent detection error.

Figure 16 and Figure 17 show the detected temperature and the percent detection error, respectively. The error follows the evolution of Figure 15, as expected, ranging within the interval ±1%. We emphasize the need for calibration to guarantee an accurate initial reading of the environment temperature. Although one expects that the sensor operation will not interfere in temperature detection (Section 2.4), the calibration should also address the assessment of the sensor with the application of a flat field temperature throughout it. This process must be repeated within the temperature detection range of the sensor. Additionally, this flat field temperature assessment could also contribute to proper mitigation of the fixed-pattern noise (FPN), which is temperature-dependent. One can design and program the sensor to operate in a differential mode: just for comparing the temperatures throughout the sensed object, for instance.

### 3.2. Frequency Response and Noise Performance

The transconductance gain of the matrix amplifier defined in Equation (6) will vary with—(i) size; (ii) the aspect ratio of all transistors (especially regarding $M_{fc}$ which is responsible for $i_{{DS}_{px}}$, and consequently, $i_{{DS}_{M}}$); (iii) the biasing voltages $V_{{Ld}_{px}}$ and $V_{{Ld}_{M}}$; and (iv) the photodiode voltage ($V_{pd}$), since it will also act as a biasing voltage for the sensing amplifier. These parameters affect not only the gain but also the bandwidth, the linearity and the linear range of sensor operation.

(6) $$G_{M}\;=\;\frac{\partial i_{{DS}_{M}}}{\partial V_{pd}}\;=\;\frac{\partial V_{{GM}_{io}}}{\partial V_{pd}}\times \frac{\partial i_{{DS}_{M}}}{\partial V_{{GM}_{io}}}.$$

**Figure 18:**
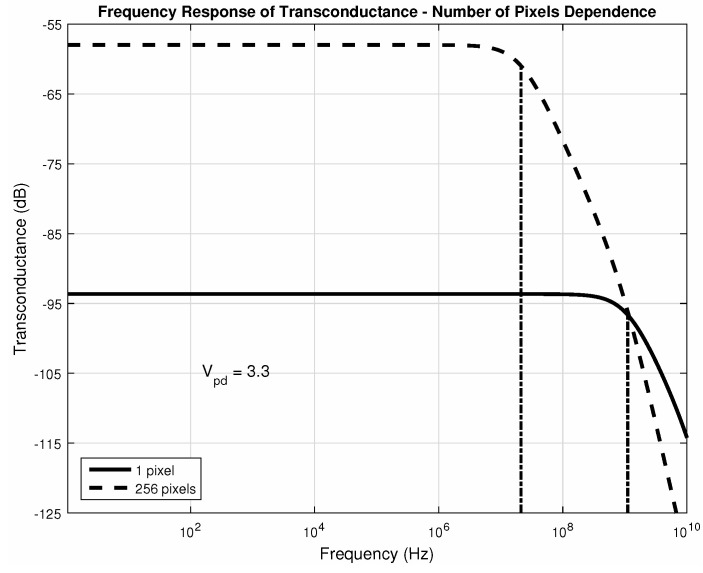
Bandwidth for different pixel numbers ($25\;{}^{\circ }C$).

The matrix size will impact the bandwidth since the capacitances of all $M_{fc}$ will be summed, and Figure 18 illustrates this. Even though each pixel is intrinsically fast, the overall matrix response is slower than just one pixel. As expected, the relationship between the gain and the bandwidth for Figure 18 is almost the same—the matrix gain is 60 times larger than that of just 1 pixel, but its bandwidth is 53 times smaller. All of the pixels were considered to be at room temperature. As mentioned before, the photodiode voltage of operation will remain closer to the reset voltage (3.3V).

**Figure 19:**
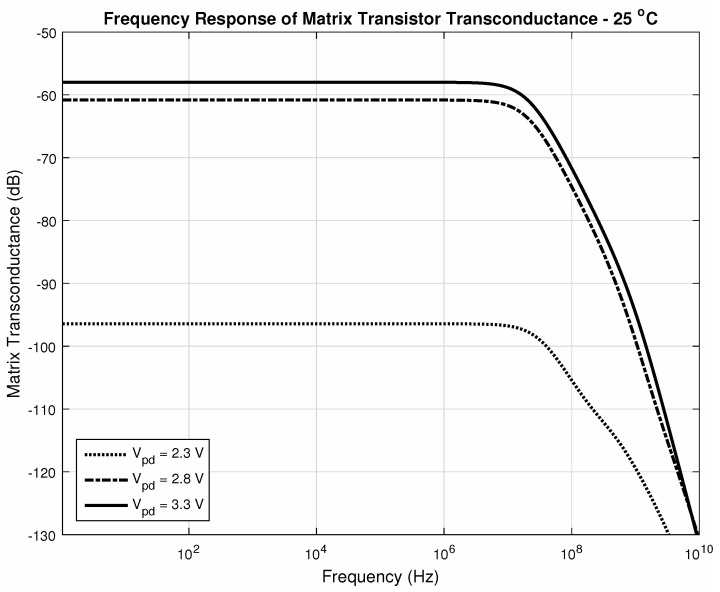
Bandwith at $25\;{}^{\circ }C$, param. $V_{pd}$.

**Figure 20:**
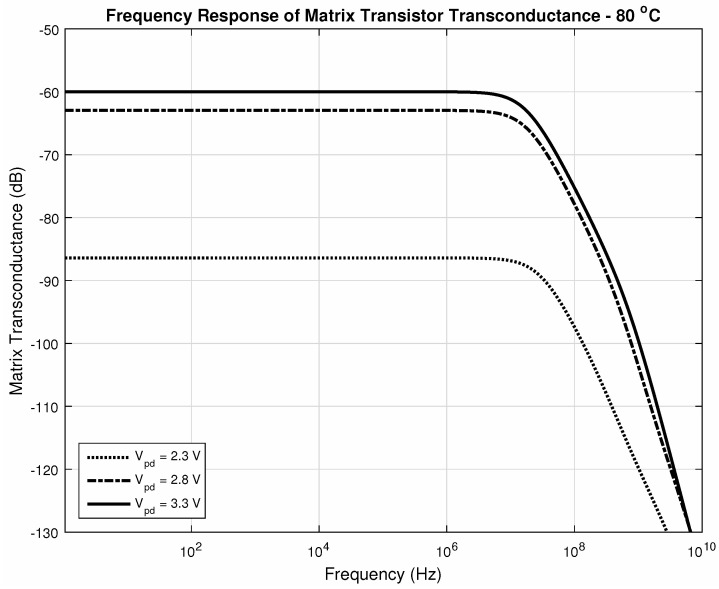
Bandwith at $80\;{}^{\circ }C$, param. $V_{pd}$.

Through Figure 19 and Figure 20, we can make a comparison between the operation at corner temperatures with different photodiode voltages. It is possible to notice the impact of the current amplification performed by the matrix. The gain-bandwidth characteristics change slightly between the two corner temperatures for higher values of photodiode voltage at its cathode. This is the expected situation since integration times are significantly short and so will be the dark current difference at each pixel in this time interval. Those changes are compensated during the $\Delta i_{{DS}_{M}}$ formation with the current subtraction, resulting in good linearity.

Figure 18, Figure 19 and Figure 20 confirm that both amplifiers, as source-followers have two poles and one zero in their transfer functions, as expected, and Table 2 summarizes the main data in those figures. Regarding all the data, we can conclude that the bandwidth is suitable for sensing temperature transients, which are in the order of tenths of milliseconds, even in the worst cases. Both gain and bandwidth change concerning the temperature [68].

It is reasonable to consider the total output noise power spectral density constant: the thermal noise is dominant at the shutter frequency of 10MHz. Based on this, it is also reasonable to put constraints on noise bandwidth: the variations of clock frequency, which acts as a carrier for the pixel information.

Table 3 presents the values for Signal-to-Noise Ratio (SNR), Dynamic Range (DR), and Noise Current. We obtained those parameters considering two scenarios at each corner temperature: (i) the best case, in which the noise is computed with a 110kHz bandwidth; and (ii) the worst case, with a 8MHz bandwidth. Both bandwidths are around 10MHz, which is the readout clock frequency. Those bandwidths were obtained from other works [70,71,72,73] regarding frequency clock variations in digital circuits designed in a CMOS process with the same feature size. The best-case value comes from thermal drift variation studies [70,71] and the worst case, from Monte Carlo analyses [72,73]. The expected performance is closer to the best scenario since the thermal drift will happen to every sensor. Monte Carlo analysis concerns the deviation in the clock frequency due to the fabrication process. Furthermore, once fabricated, the sensor will have its own performance and temperature drift.

In Table 3 we present the observed values for $i_{{DS}_{M}}$ and $\Delta i_{{DS}_{M}}$, respectively, 768.3μA and 295.9μA.

As mentioned before, regarding the operation of the sensor, $\Delta i_{{DS}_{M}}$ is obtained from the difference between the two values of $i_{{DS}_{M}}$ performing a simple form of *CDS*. This approach can improve the real sensor dynamic range since the fixed-pattern noise present at the readout will be minimized by the *CDS*. This analysis is also valid for determining the ***NETD*** (Noise Equivalent Temperature Difference)—considered to be the value of $\Delta i_{{DS}_{M}}$, which was large enough to stand out in the sensor noise floor. That would lead to a NETD<10mK, can satisfy both noise current levels, at $25\;{}^{\circ }C$ and $80\;{}^{\circ }C$, taking into account only the best case. If the shutter error is not canceled by calibration or a digital CDS, as recommended, the reevaluation would set NETD<90mK. Putting all together, as a final remark on noise performance, if the thermal contribution of the sensor is taken into account, the NETD should be at least 46mK.

Finally, considering all the discussions about the biasing voltages and the natural existence of fixed-pattern noise (FPN), although the calibration is expected to be done, FPN mitigation techniques can be implemented along with the internal CDS of the matrix to render the output results less prone to inherent process variations within the array [74].

## 4. Conclusions

It was shown that it is possible to use a CMOS APS matrix with a self-amplified output current as a contact thermal imager. We also demonstrated that it is possible to apply this concept to observe and measure temperature microgradients. The methodology may be applied to different technologies with smaller feature sizes.

We also highlight that the presented sensor is potentially cheaper than the MEMS-based ones. Moreover, this sensor, being CMOS-based and presenting no movable or suspended parts, is appropriate for monolithic integration into System-in-Package (SiP) solutions providing both compliant circuit interfaces and mechanical robustness, respectively. Also, it is fully compatible with standard processes, requiring neither design-rule violations nor customization of materials and steps in the fabrication process.

The use of current-mode operation for its readout can considerably improve the output signal swing. Although the designed pixel was made bigger than the usual 3T-APS ones, it is suitable for thermal mappings. The discussed sensor has a promising potential to perform with—(i) very low temperature errors (±1%); (ii) considerable sensitivity ($185.33\times 10^{3}\;{}^{\circ }C/\mu A$); (iii) high dynamic range and signal-to-noise ratio; (iv) low NETD; (v) high linearity; (vi) very low integration time that leads to an (vii) acceptable (30 fps) frame rate possible output.

Its utilization is suitable to situations when the contact with the object to be “monitored” is feasible and possible, and offers a good choice when a permanent, fast and integrated solution for thermal observation is desired in different situations—for thermal protection, as part of a reliability assessment system; for measurement, when minimal temperature differences are used to measure liquid or gas flow; or for monitoring, as in-vivo biological samples growing in a controlled environment, for instance.

## Figures and Tables

**Table 1:** Thermal noise generated by the sensor at $25\;{}^{\circ }C$ ^†^.

Operation Mode	Temperature Difference
Continuous Output	36mK
1 scene and rest for 1ms	1mK

^†^ Operation conditions: $\tau_{int}=10\;\mu s$, 30 fps and $f_{readout}=10\;\mathrm{MHz}$.

**Table 2:** Gain and Bandwidth for Figure 18, Figure 19 and Figure 20.

$V_{\mathrm{pd}}$	# of Pixels	Temperature ($\;\;{}^{\circ }C$)	Gain (μA/V (dB))	$f_{c}$ (MHz)
3.3V	1	25 (Figure 18)	20.78 (−93.6)	1120
	256	25 (Figure 18)	1260 (−58)	21.2
		80 (Figure 20)	1000 (−60)	17.7
2.3V	256	25 (Figure 19)	15.1 (−96.4)	35.3
		80 (Figure 20)	47.8 (−86.4)	29.9

**Table 3:** Best and Worst case for Signal-to-Noise Ratio (SNR) and Dynamic Range (DR)

	SNR (dB)		DR (dB)		Noise Current (A)	
**Temperature**	$25\;{}^{\circ }C$	$80\;{}^{\circ }C$	$25\;{}^{\circ }C$	$80\;{}^{\circ }C$	$25\;{}^{\circ }C$	$80\;{}^{\circ }C$
Best Case	84.7	84.1	76.4	75.8	44.7nA	47.9nA
Worst Case	66.1	65.5	57.8	57.2	381.4nA	408.4nA
